# The susceptibility of single nucleotide polymorphisms located within co-stimulatory pathways to systemic lupus erythematosus

**DOI:** 10.3389/fimmu.2023.1331796

**Published:** 2024-02-01

**Authors:** Ding-Ping Chen, Wei-Tzu Lin, Fang-Ping Hsu, Kuang-Hui Yu

**Affiliations:** ^1^Department of Laboratory Medicine, Linkou Chang Gung Memorial Hospital, Taoyuan, Taiwan; ^2^Department of Medical Biotechnology and Laboratory Science, College of Medicine, Chang Gung University, Taoyuan, Taiwan; ^3^Division of Rheumatology, Allergy, and Immunology, Linkou Chang Gung University and Memorial Hospital, Taoyuan, Taiwan

**Keywords:** systemic lupus erythematosus, promoter, 3 prime untranslated region, costimulatory molecules, immune regulatory genes

## Abstract

**Introduction:**

Autoimmune diseases result from the loss of immune tolerance, and they exhibit complex pathogenic mechanisms that remain challenging to effectively treat. It has been reported that the altered expression levels of co-stimulatory/inhibitory molecules will affect the level of T/B cell activation and lead to the loss of immune tolerance.

**Methods:**

In this study, we evaluated the gene polymorphisms of the ligand genes corresponding co-stimulatory system that were expressed on antigen-presenting cells (CD80, CD86, ICOSLG, and PDL1) from 60 systemic lupus erythematosus (SLE) patients and 60 healthy controls.

**Results:**

The results showed that rs16829984 and rs57271503 of the CD80 gene and rs4143815 of the PDL1 gene were associated with SLE, in which the G-allele of rs16829984 (p=0.022), the A-allele of rs57271503 (p=0.029), and the GG and GC genotype of rs4143815 (p=0.039) may be risk polymorphisms for SLE.

**Discussion:**

These SNPs are in the promoter and 3’UTR of the genes, so they may affect the transcription and translation activity of the genes, thereby regulating immune function and contributing to the development of SLE.

## Introduction

Autoimmune diseases are one of the top ten major injuries in Taiwan, their pathogenesis remains unclear up till the present moment. The widely accepted notion is that these diseases are attributed to the over-activation of autoreactive immune cells and the loss of immune tolerance (1), leading to immune responses that target and attack one’s tissues (2).

Due to the advancement of medical technology, numerous single nucleotide polymorphisms (SNPs) have been discovered to be associated with autoimmune diseases (3). In the previous study, we investigated the association between systemic lupus erythematosus (SLE) and the SNPs of the genes that were involved in regulating T-cell activation, including cytotoxic T-lymphocyte-associated protein 4 (CTLA4), CD28, programmed cell death protein 1 (PDCD1), and inducible T cell costimulator (ICOS) (4). The aforementioned genes encode co-stimulatory/inhibitory molecules expressed on the surface of T cells, which provide the necessary second signal for T-cell activation when it binds with its ligand on antigen-presenting cells (APCs). However, the activation of immune cells requires interaction between receptors and ligands to exert their effects. Therefore, further understanding is needed to determine whether the gene polymorphisms of the ligands are indeed associated with autoimmune diseases.

The CD80 (B7-1) and CD86 (B7-2) genes, located on human chromosome 3, are ligand genes for CTLA4 and CD28. They are primarily expressed on the surface of APCs, such as dendritic cells, macrophages, and B cells, and they play an important role in the immune system by interacting with CD28 and CTLA4 (5). It has been shown that increased expression of CD80 and CD86 may be involved in the presentation of autoantigens and hyperactivation of T cells, in which the immune system attacks normal tissues and cells in the body, resulting in an exaggerated immune response in autoimmune diseases (6). In recent years, significant progress has been made in the research of receptor genes CD28 and CTLA4. However, it is still a poor understanding of their ligands genes, CD80 and CD86. In certain autoimmune diseases, such as rheumatoid arthritis (RA), autoimmune thyroid disease, Graves’ ophthalmopathy (GO), and autoimmune diabetes, studies have shown that excessive expression of CD80 and CD86 may lead to overactivation of T cells and enhanced inflammatory responses, thereby promoting the development of autoimmune pathologies (7–11).

Programmed death-ligand 1 (PD-L1) usually binds with its receptor, programmed cell death protein-1 (PD-1), to form the PD-L1/PD-1 signaling pathway, which plays an important role in immune tolerance and immune regulation (12). Studies have indicated that excessive expression of PD-L1 may be involved in the occurrence and progression of autoimmune diseases, such as RA, SLE, and autoimmune diabetes (13–15). Thus, the increased expression of PD-L1 may help immune cells escape immune surveillance and inhibit T-cell activation, thereby promoting the development of autoimmune diseases. Similarly, blocking the PD-L1/PD-1 signaling pathway is an important strategy in immunotherapy for treating autoimmune diseases. By suppressing the effect of PD-L1, the attack of T cells on pathological tissues can be restored, thus inhibiting autoimmune reactions (16).

The inducible T-cell co-stimulator ligand (ICOSL) gene (ICOSLG), located on chromosome 21, is a co-stimulatory molecule, that combines with ICOS on T cells, providing a second signal to activate and regulate the function of T cells. This is important for appropriate immune response and immune tolerance. Therefore, ICOSL may be an option for the treatment of autoimmune diseases. Studies have shown that the polymorphisms of ICOSLG were associated with an increased risk of RA, autoimmune thyroid diseases, and SLE (17–19).

## Materials and methods

### The aim of this study

In our previous study, we found that the gene polymorphisms of co-stimulatory molecules expression on the surface of T cells had an association with SLE. Here, we tried to find out the association between the gene polymorphisms of the ligands located on the APC that correspond to those co-stimulatory molecules and SLE.

### The characteristics of participants

The Institutional Review Board of Chang Gung Memorial Hospital has reviewed and approved the study. The approval ID was 202002097B0. All study subjects signed informed consent and performed following relevant guidelines and regulations. In this case-control study, we evaluated 60 patients with SLE who were diagnosed based on the diagnostic criteria established by the American College of Rheumatology and 60 healthy controls without any immune abnormalities and not taking any immune-related drugs for analysis. The onset age of the SLE patients was 32.85 ± 1.76 years old, consisting of 52 females and 8 males. The average age of the SLE patients was 36.05 ± 0.87 years old, consisting of 48 females and 12 males.

### Sample collection, DNA extraction, and sequencing

The remaining samples will be extracted with the QIAamp DNA Mini kit and the QIAamp RNA Blood Mini Kit (Qiagen GmbH, Hilden, Germany) to extract DNA and RNA from the subjects. The purity and concentration of the DNA and RNA samples were evaluated by using a spectrophotometer to measure the absorbance at 260nm and 280nm. The PCR mixture contained 50ng of DNA, 7.5µl of Hotstar Taq DNA Polymerase (Qiagen GmbH, Hilden, Germany) or 2X Tag polymerase, each 1µl of forward and reverse primer (10μΜ), and 14.5µl of ddH_2_O. Then, the Big Dye Terminator Cycle Sequencing kit (Thermo Fisher, Waltham, Massachusetts, USA) and the ABI PRISM genetic analyzer (Thermo Fisher, Waltham, Massachusetts, USA) were used for direct sequencing according to the manufacturer’s instructions.

### Primer design and candidate SNP selection

Primers were designed for the SNPs located in the promoter and 3’UTR of genes that have been reported to be associated with immune-related diseases. In this experiment, we focused on the *CD80*/*CD86*, *PDL1*, and *ICOSLG*, which were the corresponding ligands for CD28/CTLA4, PD1, and ICOS, respectively. There were 43 SNPs (**Table 1**) obtained by searching the literature from NCBI. The 11 pairs of primers (**Table 2**) were designed to amplify the genomic DNA fragments covering these 43 SNPs.

**Table 1:** The 43 candidate SNP in 6 genes.

Gene	Position	SNP				
CD80	promoter	rs41271391	rs16829980	rs16829984	rs41271393	rs1880661
		rs68180496	rs139023057	rs1852212	rs3915166	rs3915165
	3UTR	rs17281703	rs1599795	rs1599796	rs113991263	rs7628626
		rs568161177	rs57271503			
CD86	promoter	rs2715267	rs114242768	rs56347468	rs2072186836	rs1378877070
	3UTR	rs1129055	rs5852291	rs1915087	rs17281995	rs1187737565
		rs995595567				
ICOSLG	promoter	rs378299	rs876200			
	3UTR	rs4819387	rs4819388	rs8130802	rs15927	rs117576800
		rs11274942				
PDL1	promoter	rs822335	rs550207667	rs822336	rs10815225	rs12342381381
	3UTR	rs4143815	rs141907112			

**Table 2:** The 11 pairs of primers for amplifying the genomic DNA fragments.

Gene	Position	Primer		Position
CD80	Promoter	F	5-GCCT TTG ATT TCA GGG TAA GAC TCC-3	119559473
		R	5-TTG CAG TGA GCC AAG ATT GCA CCA-3	119561016
	3UTR	F	5-GGG GCC AAT ATA GGG TTT GAT GTA ATC-3	119525791
		R	5-CCT GGA AGC AGT GTG AGT GTA AAT G-3	119524181
CD86	Promoter	1F	5-TGT GGA TAC GTT TTC TCT GGG C-3	122051849
		1R	5-GAA ATC CAG AAG CTG GCT AAG AG-3	122053080
		2F	5-GTT TTA TTT GCA CTC CTG CTC TGC-3	122053952
		2R	5-GGA GGA AGA AAT TTA ACC CTT TCC-3	122055291
	3’UTR	1F	5-GTT CTC TTG CTT TCT CCA CCT TTC-3	122119286
		1R	5-GCA AGC CTC CTC AAT ATT AGC AAC-3	122120254
		2F	5-TCT GGG CTG TTG CTA ATA TTG AGG-3	122120223
		2R	5-GCT TCT AGC CAT AGA GAT GTA AAG-3	122121257
ICOSL	Promoter	F	5-TAA TTC ACC TGC CCA GCC GCA T—3	44240800
		R	5-ATC TTG GTT GAA GGA ATG GCA GCC-3	44242035
	3UTR	F	5-ATT AGT GTC GCC GTC CTG CCT GT-3	44227222
		R	5-AGC GAG AAA GAC ACA GCC CAGC-3	44228265
PD-L1	promoter	1F	5-TTT GCT CAC ATC TCT TCA GGT CCA C-3	5447887
		1F	5-CTA GTT CAT GAC TCT TGA GGT CTG C-3	5448994
		2F	5-GCC ATA TGG GTC TGC TGC TGA CTT-3	5450043
		2R	5-ACT TAC TTA GAT GCT GCA GCT GGG-3	5451013
	3’UTR	F	5-CTT TTT CCC CAG ACC ACT TCC CAT G-3	5467787
		R	5-CAC ATC TGT AGA TTC AAT GCC TGG C-3	5468840

F, forward primer; R, reverse primer. Position number was obtained from Genome Assembly GRCh38.p13.

### Statistical analysis

Statistical analysis was performed using the SPSS 17.0 software, employing chi-square tests or Fisher’s exact test. In the genetic model, the genotypes (AA, Aa, and aa) were evaluated, where the allele with a higher frequency was referred to as the major allele “A,” and the other allele was referred to as the minor allele “a”. All data were analyzed using the homozygous model (AA vs. aa), heterozygous model (AA vs. Aa), dominant model (AA vs. Aa + aa), recessive model (AA + Aa vs. aa), and additive model (AA vs. Aa vs. aa) to identify significant SNPs. The allele frequencies for each SNP in the control group were in Hardy-Weinberg equilibrium (HWE). The analysis of linkage disequilibrium (LD) is performed using Haploview 4.2 software (Broad Institute, Cambridge, MA, USA; http://www.broad.mit.edu/mpg/haploview) to assess the presence of linkage relationships and conduct haplotype analysis to identify the correct pathogenic genes. For multiple comparisons, the false discovery rate (FDR) Q values were calculated to evaluate the expected proportion of Type I errors.

## Results

### The analysis of genotype frequency

The SNP analysis revealed significant findings for two SNPs of CD80: rs16829984 located in the promoter region and rs57271503 located in the 3’UTR (**Table 3**). The genotype frequency of rs16829984 had a significant difference between SLE patients and healthy controls based on the additive model (GG vs. GC vs. CC, p=0.001), recessive model (GG + GC vs. CC, p<0.001), and homozygous model (GG vs. CC, p=0.001). Additionally, the allele frequency of rs16829984 was significantly different between SLE patients and healthy controls (G vs. C, p=0.022, OR=0.495, 95% CI. = 0.279-0.878), which indicated that the C-allele of rs16829984 had a decreased risk for SLE. On the other hand, the G-allele of rs16829984 was the risk allele for SLE. The polymorphisms of rs57271503 were associated with SLE based on allele frequency (G vs. A, p=0.029, OR=1.900, 95% CI. = 1.062-3.399) and dominant model (GG vs.GA + AA, p=0.044, OR=2.104, 95% CI. = 1.015-4.361), which was suggested individuals with at least one A-allele at rs57271503 would have a higher odds of SLE.

**Table 3:** The significant SNPs that were associated with SLE.

SNP	Gene position	Minor allele no. (%)	No. of patients (%)			Allele p	OR (95% CI)			Model	Logistic regression p	OR (95% CI)			HWE	Q value
rs16829984	CD80-promoter	C	GG	GC	CC	0.022*	0.495	0.279	0.878	Additive	0.001*	NA				0.042
cases		26	34	26	0					Dominant	0.361	0.715	0.349	1.468		0.722
		38%	54%	58%	0%					Recessive	<0.001*	NA				0.010
controls		43	29	19	12					Homozygous	0.001*	NA			0.055	0.033
		62%	46%	42%	100%					Heterozygous	0.694	1.167	0.540	2.525		0.883
rs57271503	CD80-3UTR	A	GG	GA	AA	0.029*	1.900	1.062	3.399	Additive	0.085	NA				0.864
cases		40	26	28	6					Dominant	0.044*	2.104	1.015	4.361		0.685
		62%	41%	57%	75%					Recessive	0.272	3.222	0.623	16.655		1
controls		25	37	21	2					Homozygous	0.128	4.269	0.798	22.840	0.894	1
		38%	59%	43%	25%					Heterozygous	0.095	1.897	0.891	4.041		0.650
rs4143815	PD-L1-3UTR	G	CC	GC	GG	0.067	1.620	0.966	2.718	Additive	0.040*	NA				0.840
cases		57	15	33	12					Dominant	0.039*	2.300	1.036	5.108		0.685
		57%	40%	66%	48%					Recessive	0.563	0.769	0.316	1.873		1
controls		43	23	17	13					Homozygous	0.503	1.415	0.511	3.922	0.051	1
		43%	61%	34%	52%					Heterozygous	0.013*	2.976	1.241	7.140		0.273

HWE, Hardy-Weinberg equilibrium; OR, odds ratio; CI, confidence interval; NA, not applicable; *, statistical significance (p<0.05). Bold values: statistical difference (p<0.05).

In addition, one SNPs of PDL1 had statistical significance: rs4143815 in the 3’UTR (**Table 3**). The genotype frequency of rs4143815 had a significant difference between SLE patients and healthy controls based on the additive model (CC vs. GC vs. GG, p=0.040), dominant model (CC vs. GC + GG, p=0.039, OR=2.300, 95% CI. = 1.036-5.108), and heterozygous model (CC vs. GC, p=0.013, OR=2.976, 95% CI. = 1.241-7.140), which was shown that individuals with GG and GC genotypes would have higher odds of SLE compared to CC genotype. The complete data is shown in **Supplementary Table 1**.

### The analysis of linkage disequilibrium

**Figure 1:**
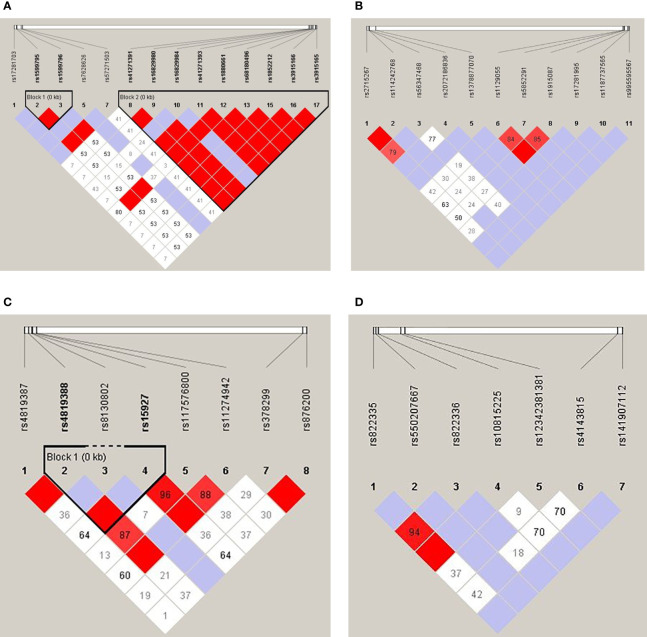
Linkage disequilibrium analysis of the CD80 **(A)**, CD86 **(B)**, ICOSLG **(C)**, and PDL1 **(D)**. The red color in the box indicates that two SNPs have strong linkage, whereas the less the linkage the closer to the white color of the box, and the light purple indicates no linkage.

Linkage disequilibrium (LD) analysis of each 4 genes was shown in **Figures 1A–D**. There were two haplotype blocks in the CD80 gene, in which one was composed of rs1599795 and rs1599796, and the other was composed of rs41271391, rs16829980, rs16829984, rs41271393, rs1880661, rs68180496, rs1852212, rs3915166, and rs3915165 (**Figure 1**), and there was one haplotype block in the ICOSLG, which was composed of rs4819388 and rs15927 (**Figure 1C**), while there was no haplotype in the CD86 and PDL1 gene.

## Discussion

In this study, we compared the SNPs in the immune-related ligand genes between 60 patients with SLE and 60 healthy controls. Because the expression level of costimulatory molecules may affect the T-cell activation level (20) and genetic variations in the promoter and 3’ UTR of genes can influence gene expression (21, 22), this project focused on investigating the promoter region and 3’ UTR of the target genes. The analysis revealed significant findings for three SNPs: rs16829984 located in the promoter region of the CD80 gene, rs57271503 located in the 3’UTR of the CD80 gene, and rs4143815 in the 3’UTR of the PDL1 gene.

The CD80 gene is an important immunoregulatory gene that plays a crucial role in the human immune system. The protein encoded by the CD80 gene (CD80 molecule) is a co-stimulatory molecule, also known as B7.1, which is mainly expressed on the surface of antigen-presenting cells such as dendritic cells, macrophages, and B cells. After binding to CD28, the CD28-CD80 pathway provides an important costimulatory signal to activate and regulate the immune response of T cells. This signal is essential for T-cell activation, leading to T-cell proliferation, differentiation, and the release of immune factors to combat infection or pathological conditions. Thus, abnormal expression levels and functional alterations of CD80 are closely associated with various immune-related disorders, including autoimmune diseases, allergic reactions, and tumor immune evasion (23). Studies have shown that modulating the CD80 signaling pathway may hold significant importance in regulating immune system balance, treating certain diseases, and developing immunotherapies (24). This study found that the G-allele of rs16829984 and the A-allele of rs57271503 may be risk alleles for SLE. Additionally, the related studies have shown that rs16829984 was statistically associated with cervical cancer, while rs57271503 is statistically associated with rheumatoid arthritis (25, 26). Consequently, these genetic variations have susceptibility to diseases that may be due to the influence of gene transcription or translation. However, there is limited literature available on these two SNPs at present and the bio-function of these SNPs needs to be further explored. In addition, our published findings showed that several SNPs in the promoter region of CTLA4 were associated with SLE, while there was no susceptibility SNP in the CD28 gene (4). Integrating with the results of this project, it was suggested that the CD80/CTLA4 pathway, namely the inhibiting signal, may be more important for the development of SLE than the active pathway.

The PDL1 gene also plays a crucial role in the human immune system. PD-L1 is an immune checkpoint molecule that primarily functions to regulate the balance of immune responses and maintain the self-regulatory mechanisms of the immune system. The primary ligand for PD-L1 is the PD-1 receptor, which is typically expressed on activated T cells, B cells, and other immune cells. When PD-L1 binds to PD-1, it inhibits the activation and effector functions of T cells, which is crucial for maintaining immune tolerance and preventing excessive immune responses (27). In certain disease conditions, such as tumors and infections, tumor cells or pathogens may overexpress PD-L1. This overexpression can lead to the formation of immune evasion mechanisms, allowing the tumor or pathogen to evade attacks from the immune system (28–30). This study found that the rs4143815 variant located in the 3’UTR of the PD-L1 gene is statistically associated with SLE. Individuals with the GG or GC genotypes have a higher odds ratio of developing SLE. The polymorphism of this SNP was also associated with various diseases. For example, GG genotype was associated with poor prognosis in lung adenocarcinoma and squamous cell carcinoma (31), the CC genotype was associated with a two times higher risk of developing squamous cell lung cancer (32), G-allele was associated with poor outcomes in the early stage non-small cell lung cancer (33), the CC genotype has significantly reduced the risk of breast cancer (34), individuals carrying the C-allele of rs4143815 had less risk of type 1 diabetes (35), and so on. In addition, a functional study has already demonstrated that the rs4143815 C>G variant reduced transcriptional activity of the PDL1 gene (33), which indicated that the statistical association between rs4143815 and the diseases may be due to the gene expression level alteration caused by SNP variation, which in turn contributes to the development of the diseases.

Although the SNPs in non-coding gene regions do not directly affect protein function and structure, they play a crucial role in regulating gene expression (36). Gene variations in the promoter region may affect the initiation and activity of transcription through interactions with transcription factors, DNA methylation, or serving as targets for miRNA and other epigenetic mechanisms (21), and the sequence of the 3’UTR plays a crucial role in processes such as mRNA stability, localization, translational activity, pre-mRNA processing, and subsequent protein synthesis (22), leading to a significant association between these non-coding gene SNPs and numerous complex diseases. However, many SNP variations are neutral, namely they have no impact on biological function. Therefore, the functional effect of these disease susceptibility SNPs needs to be further explored in the future and it can be assisted by using silico analysis to predict transcription factor binding sites, performing expression quantitative trait loci analysis to understand the association between genetic variants and gene expression levels, and so on. At the gene level, it can only be used to predict the odds of disease. Moreover, the mRNA levels altered by non-coding gene SNPs are not a reliable indicator of corresponding protein abundance (37). To accurately reflect the actual protein expression, the use of flow cytometry to detect protein expression is also necessary.

According to current research literature, autoimmune diseases are usually complex and multifactorial, making it difficult to accurately determine their course and effectively treat them. Clinical experience has shown that the same medication may be effective for some individuals while ineffective for others, leading to time-consuming trial-and-error processes. Therefore, it is hoped that through this project, key mechanisms of autoimmune diseases can be identified, and personalized medicine can be achieved by selecting the most suitable medication based on genetic markers specific to each patient. Alternatively, the research findings from this project can provide new directions for the treatment of autoimmune diseases. It is worth noting that the mechanism of autoimmune diseases is a complex disease process that involves the interaction of numerous genes and immune regulatory factors. The role of the genes encoding co-stimulatory molecules and their ligands may only be the tip of the iceberg, and further research is still required to fully understand its specific role in autoimmune diseases.

In conclusion, the findings of this study revealed the association between gene polymorphisms of co-stimulatory ligand genes and SLE. However, these disease-associated SNP findings can only be used for statistical correlation to aid in clinical predictions and cannot explain direct causal relationships between SNPs and diseases. Therefore, in the future, it will be necessary to provide evidence of the correlation between gene variations and protein expression to elucidate the mechanisms by which the SNP affects the disease.

## Data availability statement

The datasets presented in this study can be found in online repositories. The names of the repository/repositories and accession number(s) can be found below: SS2137544506 and SS6403964567 - SS6403964612 (dbSNP; (https://www.ncbi.nlm.nih.gov/SNP/snp_viewBatch.cgi?sbid=1063574).

## Ethics statement

The studies involving humans were approved by The Institutional Review Board of Chang Gung Memorial Hospital has reviewed and approved the study. The approval ID was 202002097B0 and 202102018B0C601. The studies were conducted in accordance with the local legislation and institutional requirements. The participants provided their written informed consent to participate in this study.

## Author contributions

D-PC: Conceptualization, Funding acquisition, Writing – review & editing. W-TL: Data curation, Formal analysis, Methodology, Writing – original draft. F-PH: Data curation, Formal analysis, Methodology, Writing – original draft. K-HY: Resources, Writing – review & editing.
