# Supplementary material for: The susceptibility of single nucleotide polymorphisms located within co-stimulatory pathways to systemic lupus erythematosus
Source: Front Immunol. 2024 Feb 1;14:1331796. doi: 10.3389/fimmu.2023.1331796 (PMC10867627; doi:10.3389/fimmu.2023.1331796)
Supplement: Supplementary file 1 [file Table_1.docx]

Supplementary Table 1. The complete data of SNP analysis

| SNP | Gene position | minor allele no. (%) | No. of patients (%) | | | Allele p-value | OR (95 % CI) | | | Model | Logistic regression p-value |  | 95 % CI) | | | HWE p-value |
| --- | --- | --- | --- | --- | --- | --- | --- | --- | --- | --- | --- | --- | --- | --- | --- | --- |
|  |  |  |  |  |  |  | value | lower | upper |  |  | OR | | lower | upper |  |
| rs41271391 | CD80 promoter | G | GG | GT | TT | 0.195 | 0.729 | 0.451 | 1.177 | Additive | 0.131 | NA | |  |  |  |
| test |  | 86 | 29 | 28 | 3 |  |  |  |  | Dominant | 0.200 | 0.624 | | 0.303 | 1.285 |  |
|  |  | 59% | 45% | 54% | 100% |  |  |  |  | Recessive | 0.244 | NA | |  |  |  |
| control |  | 96 | 36 | 24 | 0 |  |  |  |  | Homozygous | 0.099 | NA | |  |  | 0.153 |
|  |  | 67% | 55% | 46% | 0% |  |  |  |  | Heterozygous | 0.321 | 0.690 | | 0.332 | 1.436 |  |
|  |  |  |  |  |  |  |  |  |  |  |  |  | |  |  |  |
| rs16829980 | CD80 promoter | A | AA | AG | GG | 0.245 | 0.752 | 0.465 | 1.217 | Additive | 0.535 | NA | |  |  |  |
| test |  | 86 | 29 | 28 | 3 |  |  |  |  | Dominant | 0.272 | 0.668 | | 0.325 | 1.374 |  |
|  |  | 59% | 45% | 55% | 60% |  |  |  |  | Recessive | 1.000 | 1.526 | | 0.246 | 9.478 |  |
| control |  | 93 | 35 | 23 | 2 |  |  |  |  | Homozygous | 0.657 | 0.552 | | 0.086 | 3.533 | 0.745 |
|  |  | 66% | 55% | 45% | 40% |  |  |  |  | Heterozygous | 0.307 | 0.681 | | 0.325 | 1.425 |  |
|  |  |  |  |  |  |  |  |  |  |  |  |  | |  |  |  |
| rs16829984 | CD80 promoter | G | GG | GC | CC | 0.523 | 1.174 | 0.718 | 1.919 | Additive | **0.001** | NA | |  |  |  |
| test |  | 94 | 34 | 26 | 0 |  |  |  |  | Dominant | 0.361 | 1.398 | | 0.681 | 2.869 |  |
|  |  | 55% | 54% | 58% | 0% |  |  |  |  | Recessive | **3E-04** | NA | |  |  |  |
| control |  | 77 | 29 | 19 | 12 |  |  |  |  | Homozygous | **0.001** | NA | |  |  | 0.055 |
|  |  | 45% | 46% | 42% | 100% |  |  |  |  | Heterozygous | 0.694 | 0.857 | | 0.396 | 1.853 |  |
|  |  |  |  |  |  |  |  |  |  |  |  |  | |  |  |  |
| rs41271393 | CD80 promoter | C | CC | CT | TT | 0.171 | 0.713 | 0.440 | 1.157 | Additive | 0.433 | NA | |  |  |  |
| test |  | 86 | 29 | 28 | 3 |  |  |  |  | Dominant | 0.200 | 0.624 | | 0.303 | 1.285 |  |
|  |  | 48% | 45% | 56% | 60% |  |  |  |  | Recessive | 1.000 | 1.526 | | 0.246 | 9.478 |  |
| control |  | 94 | 36 | 22 | 2 |  |  |  |  | Homozygous | 0.654 | 0.537 | | 0.084 | 3.432 | 0.825 |
|  |  | 52% | 55% | 44% | 40% |  |  |  |  | Heterozygous | 0.226 | 0.633 | | 0.301 | 1.330 |  |
|  |  |  |  |  |  |  |  |  |  |  |  |  | |  |  |  |
| rs1880661 | CD80 promoter | A | AA | AG | GG | 0.162 | 1.413 | 0.870 | 2.296 | Additive | 0.411 | NA | |  |  |  |
| test |  | 91 | 35 | 21 | 4 |  |  |  |  | Dominant | 0.201 | 1.600 | | 0.778 | 3.292 |  |
|  |  | 52% | 56% | 43% | 50% |  |  |  |  | Recessive | 1.000 | 0.800 | | 0.184 | 3.487 |  |
| control |  | 84 | 28 | 28 | 4 |  |  |  |  | Homozygous | 1.000 | 1.250 | | 0.287 | 5.449 | 0.690 |
|  |  | 48% | 44% | 57% | 50% |  |  |  |  | Heterozygous | 0.182 | 1.667 | | 0.785 | 3.539 |  |
|  |  |  |  |  |  |  |  |  |  |  |  |  | |  |  |  |
| rs68180496 | CD80 promoter | G | GG | GT | TT | 0.217 | 0.737 | 0.454 | 1.197 | Additive | 0.426 | NA | |  |  |  |
| test |  | 86 | 29 | 28 | 3 |  |  |  |  | Dominant | 0.272 | 0.668 | | 0.325 | 1.374 |  |
|  |  | 49% | 45% | 57% | 43% |  |  |  |  | Recessive | 1.000 | 0.737 | | 0.158 | 3.443 |  |
| control |  | 91 | 35 | 21 | 4 |  |  |  |  | Homozygous | 1.000 | 1.105 | | 0.229 | 5.341 | 0.941 |
|  |  | 51% | 55% | 43% | 57% |  |  |  |  | Heterozygous | 0.213 | 0.621 | | 0.294 | 1.315 |  |
|  |  |  |  |  |  |  |  |  |  |  |  |  | |  |  |  |
| rs139023057 | CD80 promoter | ins | ins | insTCATG | TCATG | 0.352 | 0.796 | 0.493 | 1.287 | Additive | 0.538 | NA | |  |  |  |
| test |  | 85 | 29 | 27 | 4 |  |  |  |  | Dominant | 0.361 | 0.715 | | 0.349 | 1.468 |  |
|  |  | 48% | 46% | 53% | 67% |  |  |  |  | Recessive | 0.679 | 2.071 | | 0.365 | 11.762 |  |
| control |  | 92 | 34 | 24 | 2 |  |  |  |  | Homozygous | 0.416 | 0.426 | | 0.073 | 2.499 | 0.658 |
|  |  | 52% | 54% | 47% | 33% |  |  |  |  | Heterozygous | 0.463 | 0.758 | | 0.362 | 1.589 |  |
|  |  |  |  |  |  |  |  |  |  |  |  |  | |  |  |  |
| rs1852212 | CD80 promoter | C | CC | CT | TT | 0.171 | 0.713 | 0.440 | 1.157 | Additive | 0.433 | NA | |  |  |  |
| test |  | 86 | 29 | 28 | 3 |  |  |  |  | Dominant | 0.200 | 0.624 | | 0.303 | 1.285 |  |
|  |  | 48% | 45% | 56% | 60% |  |  |  |  | Recessive | 1.000 | 1.526 | | 0.246 | 9.478 |  |
| control |  | 94 | 36 | 22 | 2 |  |  |  |  | Homozygous | 0.654 | 0.537 | | 0.084 | 3.432 | 0.825 |
|  |  | 52% | 55% | 44% | 40% |  |  |  |  | Heterozygous | 0.226 | 0.633 | | 0.301 | 1.330 |  |
|  |  |  |  |  |  |  |  |  |  |  |  |  | |  |  |  |
| rs3915166 | CD80 promoter | A | AA | AG | GG | 0.171 | 0.713 | 0.440 | 1.157 | Additive | 0.433 | NA | |  |  |  |
| test |  | 86 | 29 | 28 | 3 |  |  |  |  | Dominant | 0.200 | 0.624 | | 0.303 | 1.285 |  |
|  |  | 48% | 45% | 56% | 60% |  |  |  |  | Recessive | 1.000 | 1.526 | | 0.246 | 9.478 |  |
| control |  | 94 | 36 | 22 | 2 |  |  |  |  | Homozygous | 0.654 | 0.537 | | 0.084 | 3.432 | 0.825 |
|  |  | 52% | 55% | 44% | 40% |  |  |  |  | Heterozygous | 0.226 | 0.633 | | 0.301 | 1.330 |  |
|  |  |  |  |  |  |  |  |  |  |  |  |  | |  |  |  |
| rs3915165 | CD80 promoter | G | GG | GT | TT | 0.171 | 0.713 | 0.440 | 1.157 | Additive | 0.433 | NA | |  |  |  |
| test |  | 86 | 29 | 28 | 3 |  |  |  |  | Dominant | 1.000 | 0.655 | | 0.106 | 4.069 |  |
|  |  | 48% | 45% | 56% | 60% |  |  |  |  | Recessive | 1.000 | 1.526 | | 0.246 | 9.478 |  |
| control |  | 94 | 36 | 22 | 2 |  |  |  |  | Homozygous | 0.654 | 0.537 | | 0.084 | 3.432 | 0.825 |
|  |  | 52% | 55% | 44% | 40% |  |  |  |  | Heterozygous | 0.226 | 0.633 | | 0.301 | 1.330 |  |
|  |  |  |  |  |  |  |  |  |  |  |  |  | |  |  |  |
| rs57271503 | CD80 3UTR | G | GG | GA | AA | **0.038** | 0.598 | 0.367 | 0.973 | Additive | 0.085 | N/A | |  |  |  |
| test |  | 80 | 26 | 28 | 6 |  |  |  |  | Dominant | **0.044** | 0.475 | | 0.229 | 0.986 |  |
|  |  | 46% | 41% | 57% | 75% |  |  |  |  | Recessive | 0.272 | 3.222 | | 0.623 | 16.655 |  |
| control |  | 95 | 37 | 21 | 2 |  |  |  |  | Homozygous | 0.128 | 0.234 | | 0.044 | 1.253 | 0.990 |
|  |  | 54% | 59% | 43% | 25% |  |  |  |  | Heterozygous | 0.095 | 0.527 | | 0.247 | 1.123 |  |
|  |  |  |  |  |  |  |  |  |  |  |  |  | |  |  |  |
| rs568161177 | CD80 3UTR | A | AA | AC | CC | 0.498 | N/A |  |  | Additive | 0.315 | N/A | |  |  |  |
| test |  | 120 | 60 | 0 | 0 |  |  |  |  | Dominant | 1.000 | N/A | |  |  |  |
|  |  | 50% | 50% | 0% | 0% |  |  |  |  | Recessive | N/A | N/A | |  |  |  |
| control |  | 119 | 59 | 1 | 0 |  |  |  |  | Homozygous | N/A | N/A | |  |  | 0.998 |
|  |  | 50% | 50% | 100% | 0% |  |  |  |  | Heterozygous | 1.000 | N/A | |  |  |  |
|  |  |  |  |  |  |  |  |  |  |  |  |  | |  |  |  |
| rs7628626 | CD80 3UTR | C | CC | AC | AA | 1.000 | 1.000 | 0.567 | 1.764 | Additive | 1.000 | N/A | |  |  |  |
| test |  | 103 | 44 | 15 | 1 |  |  |  |  | Dominant | 1.000 | 1.000 | | 0.445 | 2.246 |  |
|  |  | 50% | 50% | 50% | 50% |  |  |  |  | Recessive | 1.000 | 1.000 | | 0.061 | 16.366 |  |
| control |  | 103 | 44 | 15 | 1 |  |  |  |  | Homozygous | 1.000 | 1.000 | | 0.061 | 16.496 | 0.997 |
|  |  | 50% | 50% | 50% | 50% |  |  |  |  | Heterozygous | 1.000 | 1.000 | | 0.437 | 2.290 |  |
|  |  |  |  |  |  |  |  |  |  |  |  |  | |  |  |  |
| rs113991263 | CD80 3UTR | TCT | TCT | delTCT | del | 0.498 | N/A |  |  | Additive | 0.315 | N/A | |  |  |  |
| test |  | 120 | 60 | 0 | 0 |  |  |  |  | Dominant | 1.000 | N/A | |  |  |  |
|  |  | 50% | 50% | 0% | 0% |  |  |  |  | Recessive | N/A | N/A | |  |  |  |
| control |  | 119 | 59 | 1 | 0 |  |  |  |  | Homozygous | N/A | N/A | |  |  | 0.998 |
|  |  | 50% | 50% | 100% | 0% |  |  |  |  | Heterozygous | 1.000 | N/A | |  |  |  |
|  |  |  |  |  |  |  |  |  |  |  |  |  | |  |  |  |
| rs1599796 | CD80 3UTR | G | GG | GA | AA | 0.325 | 1.274 | 0.786 | 2.064 | Additive | 0.627 | N/A | |  |  |  |
| test |  | 86 | 32 | 22 | 6 |  |  |  |  | Dominant | 0.361 | 1.397 | | 0.681 | 2.865 |  |
|  |  | 51% | 54% | 45% | 50% |  |  |  |  | Recessive | 1.000 | 1.000 | | 0.303 | 3.296 |  |
| control |  | 81 | 27 | 27 | 6 |  |  |  |  | Homozygous | 0.788 | 1.185 | | 0.342 | 4.104 | 1.000 |
|  |  | 49% | 46% | 55% | 50% |  |  |  |  | Heterozygous | 0.334 | 1.455 | | 0.680 | 3.114 |  |
|  |  |  |  |  |  |  |  |  |  |  |  |  | |  |  |  |
| rs1599795 | CD80 3UTR | T | TT | TA | AA | 0.244 | 1.335 | 0.820 | 2.173 | Additive | 0.539 | N/A | |  |  |  |
| test |  | 86 | 32 | 22 | 6 |  |  |  |  | Dominant | 0.266 | 1.509 | | 0.730 | 3.118 |  |
|  |  | 53% | 56% | 46% | 46% |  |  |  |  | Recessive | 0.720 | 0.810 | | 0.255 | 2.571 |  |
| control |  | 76 | 25 | 26 | 7 |  |  |  |  | Homozygous | 0.514 | 1.493 | | 0.446 | 5.005 | 0.992 |
|  |  | 47% | 44% | 54% | 54% |  |  |  |  | Heterozygous | 0.292 | 1.513 | | 0.699 | 3.274 |  |
|  |  |  |  |  |  |  |  |  |  |  |  |  | |  |  |  |
| rs17281703 | CD80 3UTR | G | GG | GA | AA | 0.247 | 1.529 | 0.743 | 3.146 | Additive | 0.402 | N/A | |  |  |  |
| test |  | 112 | 52 | 8 | 0 |  |  |  |  | Dominant | 0.322 | 1.667 | | 0.602 | 4.614 |  |
|  |  | 56% | 57% | 47% | 0% |  |  |  |  | Recessive | 0.450 | N/A | |  |  |  |
| control |  | 87 | 39 | 9 | 1 |  |  |  |  | Homozygous | 0.435 | N/A | |  |  | 0.850 |
|  |  | 44% | 43% | 53% | 100% |  |  |  |  | Heterozygous | 0.442 | 1.500 | | 0.531 | 4.239 |  |
|  |  |  |  |  |  |  |  |  |  |  |  |  | |  |  |  |
| rs2715267 | CD86 promoter | T | TT | GT | GG | 0.155 | 1.526 | 0.850 | 2.739 | Additive | 0.326 | N/A | |  |  |  |
| test |  | 93 | 35 | 23 | 2 |  |  |  |  | Recessive | 0.431 | 0.457 | | 0.080 | 2.597 |  |
|  |  | 54% | 57% | 46% | 33% |  |  |  |  | Dominant | 0.169 | 1.669 | | 0.803 | 3.469 |  |
| control |  | 79 | 26 | 27 | 4 |  |  |  |  | Heterozygous | 0.232 | 1.580 | | 0.744 | 3.355 | 0.694 |
|  |  | 46% | 43% | 54% | 67% |  |  |  |  | Homozygous | 0.396 | 2.692 | | 0.458 | 15.832 |  |
|  |  |  |  |  |  |  |  |  |  |  |  |  | |  |  |  |
| rs114242768 | CD86 promoter | C | CC | CT | TT | 0.974 | 1.018 | 0.346 | 2.997 | Additive | 1.000 | N/A | |  |  |  |
| test |  | 113 | 54 | 5 | 1 |  |  |  |  | Recessive | 1.000 | 0.983 | | 0.060 | 16.092 |  |
|  |  | 50% | 50% | 50% | 50% |  |  |  |  | Dominant | 0.976 | 1.019 | | 0.309 | 3.360 |  |
| control |  | 111 | 53 | 5 | 1 |  |  |  |  | Heterozygous | 1.000 | 1.019 | | 0.279 | 3.724 | 0.181 |
|  |  | 50% | 50% | 50% | 50% |  |  |  |  | Homozygous | 0.976 | 1.019 | | 0.309 | 3.360 |  |
|  |  |  |  |  |  |  |  |  |  |  |  |  | |  |  |  |
| rs56347468 | CD86 promoter | C | CC | CG | GG | 0.180 | 1.536 | 0.818 | 2.885 | Additive | 0.362 | N/A | |  |  |  |
| test |  | 99 | 40 | 19 | 1 |  |  |  |  | Recessive | 0.619 | 0.483 | | 0.043 | 5.475 |  |
|  |  | 53% | 56% | 43% | 33% |  |  |  |  | Dominant | 0.166 | 1.688 | | 0.804 | 3.544 |  |
| control |  | 89 | 32 | 25 | 2 |  |  |  |  | Heterozygous | 0.196 | 1.645 | | 0.772 | 3.504 | 0.547 |
|  |  | 47% | 44% | 57% | 67% |  |  |  |  | Homozygous | 0.587 | 2.500 | | 0.217 | 28.828 |  |
|  |  |  |  |  |  |  |  |  |  |  |  |  | |  |  |  |
| rs2072186836 | CD86 promoter | C | CC | CG | GG | 0.224 | 2.109 | 0.618 | 7.203 | Additive | 0.212 | 2.196 | | 0.624 | 7.732 |  |
| test |  | 116 | 56 | 4 | 0 |  |  |  |  | Recessive | N/A | N/A | |  |  |  |
|  |  | 51% | 52% | 33% | 0% |  |  |  |  | Dominant | 0.212 | 2.196 | | 0.624 | 7.732 |  |
| control |  | 110 | 51 | 8 | 0 |  |  |  |  | Heterozygous | 0.212 | 2.196 | | 0.624 | 7.732 | 0.856 |
|  |  | 49% | 48% | 67% | 0% |  |  |  |  | Homozygous | N/A | N/A | |  |  |  |
|  |  |  |  |  |  |  |  |  |  |  |  |  | |  |  |  |
| rs1378877070 | CD86 promoter | TTTA | TTTA | TTTADEL | DEL | 1.000 | 1.139 | 0.0703 | 18.439 | Additive | 0.927 | 1.140 | | 0.0695 | 18.7039 |  |
| test |  | 115 | 57 | 1 | 0 |  |  |  |  | Dominant | 1.000 | 1.140 | | 0.0695 | 18.7039 |  |
|  |  | 53% | 53% | 50% | 0% |  |  |  |  | Recessive | N/A | N/A | |  |  |  |
| control |  | 101 | 50 | 1 | 0 |  |  |  |  | Homozygous | N/A | N/A | |  |  | 0.998 |
|  |  | 47% | 47% | 50% | 0% |  |  |  |  | Heterozygous | 1.000 | 1.140 | | 0.0695 | 18.7039 |  |
|  |  |  |  |  |  |  |  |  |  |  |  |  | |  |  |  |
| rs1129055 | CD86 3UTR | A | AA | GA | GG | 0.412 | 0.804 | 0.478 | 1.353 | Additive | 0.658 | NA | |  |  |  |
| test |  | 69 | 18 | 33 | 9 |  |  |  |  | Dominant | 0.516 | 0.776 | | 0.360 | 1.671 |  |
|  |  | 48% | 46% | 51% | 60% |  |  |  |  | Recessive | 0.427 | 1.559 | | 0.518 | 4.693 |  |
| control |  | 74 | 21 | 32 | 6 |  |  |  |  | Homozygous | 0.362 | 0.571 | | 0.170 | 1.916 | 0.997 |
|  |  | 52% | 54% | 49% | 40% |  |  |  |  | Heterozygous | 0.649 | 0.831 | | 0.375 | 1.841 |  |
|  |  |  |  |  |  |  |  |  |  |  |  |  | |  |  |  |
| rs5852291 | CD86 3UTR | DEL | DEL | DEL/TT | TT | 0.575 | 0.862 | 0.512 | 1.451 | Additive | 0.679 | NA | |  |  |  |
| test |  | 71 | 17 | 37 | 6 |  |  |  |  | Dominant | 0.396 | 0.715 | | 0.330 | 1.552 |  |
|  |  | 49% | 45% | 54% | 50% |  |  |  |  | Recessive | 0.976 | 0.981 | | 0.298 | 3.237 |  |
| control |  | 74 | 21 | 32 | 6 |  |  |  |  | Homozygous | 0.750 | 0.810 | | 0.221 | 2.970 | 0.471 |
|  |  | 51% | 55% | 46% | 50% |  |  |  |  | Heterozygous | 0.379 | 0.700 | | 0.316 | 1.551 |  |
|  |  |  |  |  |  |  |  |  |  |  |  |  | |  |  |  |
| rs1915087 | CD86 3UTR | C | CC | TC | TT | 0.670 | 0.893 | 0.531 | 1.503 | Additive | 0.895 | NA | |  |  |  |
| test |  | 71 | 19 | 33 | 8 |  |  |  |  | Dominant | 0.650 | 0.839 | | 0.392 | 1.796 |  |
|  |  | 49% | 48% | 52% | 53% |  |  |  |  | Recessive | 0.809 | 1.143 | | 0.386 | 3.381 |  |
| control |  | 73 | 21 | 31 | 7 |  |  |  |  | Homozygous | 0.700 | 0.792 | | 0.241 | 2.600 | 0.684 |
|  |  | 51% | 53% | 48% | 47% |  |  |  |  | Heterozygous | 0.687 | 0.850 | | 0.385 | 1.874 |  |
|  |  |  |  |  |  |  |  |  |  |  |  |  | |  |  |  |
| rs17281995 | CD86 3UTR | G | GG | GC | CC | 0.815 | 0.852 | 0.223 | 3.256 | Additive | 0.811 | 0.846 | | 0.215 | 3.324 |  |
| test |  | 115 | 55 | 5 | 0 |  |  |  |  | Dominant | 1.000 | 0.846 | | 0.215 | 3.324 |  |
|  |  | 52% | 51% | 56% | 0% |  |  |  |  | Recessive | NA | NA | |  |  |  |
| control |  | 108 | 52 | 4 | 0 |  |  |  |  | Homozygous | NA | NA | |  |  | 0.962 |
|  |  | 48% | 49% | 44% | 0% |  |  |  |  | Heterozygous | 1.000 | 0.846 | | 0.215 | 3.324 |  |
|  |  |  |  |  |  |  |  |  |  |  |  |  | |  |  |  |
| rs995595567 | CD86 3UTR | T | TT | TC | CC | 0.531 | 2.127 | 0.190 | 23.792 | Additive | 0.528 | 2.148 | | 0.189 | 24.374 |  |
| test |  | 117 | 58 | 1 | 0 |  |  |  |  | Dominant | 0.612 | 2.148 | | 0.189 | 24.374 |  |
|  |  | 52% | 52% | 33% | 0% |  |  |  |  | Recessive | NA | NA | |  |  |  |
| control |  | 110 | 54 | 2 | 0 |  |  |  |  | Homozygous | NA | NA | |  |  | 0.991 |
|  |  | 48% | 48% | 67% | 0% |  |  |  |  | Heterozygous | 0.612 | 2.148 | | 0.189 | 24.374 |  |
|  |  |  |  |  |  |  |  |  |  |  |  |  | |  |  |  |
| rs378299 | ICOSLG-promoter | T | TT | CT | CC | 0.525 | 1.183 | 0.704 | 1.986 | Additive | 0.773 | NA | |  |  |  |
| test |  | 74 | 22 | 30 | 8 |  |  |  |  | Dominant | 0.477 | 1.319 | | 0.615 | 2.829 |  |
|  |  | 52% | 55% | 48% | 47% |  |  |  |  | Recessive | 0.765 | 0.855 | | 0.306 | 2.390 |  |
| control |  | 68 | 18 | 32 | 9 |  |  |  |  | Homozygous | 0.583 | 1.375 | | 0.441 | 4.291 | 0.768 |
|  |  | 48% | 45% | 52% | 53% |  |  |  |  | Heterozygous | 0.514 | 1.304 | | 0.587 | 2.894 |  |
|  |  |  |  |  |  |  |  |  |  |  |  |  | |  |  |  |
| rs876200 | ICOSLG-promoter | C | CC | CT | TT | 0.880 | 0.947 | 0.471 | 1.907 | Additive | 0.570 | NA | |  |  |  |
| test |  | 91 | 35 | 21 | 1 |  |  |  |  | Dominant | 0.641 | 0.823 | | 0.362 | 1.869 |  |
|  |  | 56% | 55% | 62% | 33% |  |  |  |  | Recessive | 0.579 | 0.375 | | 0.033 | 4.275 |  |
| control |  | 71 | 29 | 13 | 2 |  |  |  |  | Homozygous | 0.592 | 2.414 | | 0.208 | 27.983 | 0.954 |
|  |  | 44% | 45% | 38% | 67% |  |  |  |  | Heterozygous | 0.500 | 0.747 | | 0.320 | 1.746 |  |
|  |  |  |  |  |  |  |  |  |  |  |  |  | |  |  |  |
| rs4819387 | ICOSLG, 3UTR | C | CC | CT | TT | 0.180 | 1.684 | 0.782 | 3.626 | Additive | 0.330 | NA | |  |  |  |
| test |  | 107 | 48 | 11 | 1 |  |  |  |  | Dominant | 0.142 | 1.889 | | 0.802 | 4.446 |  |
|  |  | 55% | 57% | 41% | 50% |  |  |  |  | Recessive | 1.000 | 0.881 | | 0.054 | 14.447 |  |
| control |  | 88 | 36 | 16 | 1 |  |  |  |  | Homozygous | 1.000 | 1.333 | | 0.081 | 22.043 | 0.876 |
|  |  | 45% | 43% | 59% | 50% |  |  |  |  | Heterozygous | 0.137 | 1.939 | | 0.804 | 4.680 |  |
|  |  |  |  |  |  |  |  |  |  |  |  |  | |  |  |  |
| rs4819388 | ICOSLG, 3UTR | C | CC | TC | TT | 0.688 | 1.116 | 0.652 | 1.912 | Additive | 0.935 | NA | |  |  |  |
| test |  | 72 | 22 | 28 | 10 |  |  |  |  | Dominant | 0.714 | 1.158 | | 0.529 | 2.536 |  |
|  |  | 55% | 56% | 53% | 53% |  |  |  |  | Recessive | 0.891 | 0.933 | | 0.347 | 2.511 |  |
| control |  | 59 | 17 | 25 | 9 |  |  |  |  | Homozygous | 0.786 | 1.165 | | 0.387 | 3.501 | 0.999 |
|  |  | 45% | 44% | 47% | 47% |  |  |  |  | Heterozygous | 0.733 | 1.155 | | 0.503 | 2.654 |  |
|  |  |  |  |  |  |  |  |  |  |  |  |  | |  |  |  |
| rs8130802 | ICOSLG, 3UTR | C | CC | CT | TT | 0.915 | 1.113 | 0.154 | 8.042 | Additive | 0.915 | 1.115 | | 0.152 | 8.204 |  |
| test |  | 118 | 58 | 2 | 0 |  |  |  |  | Dominant | 1.000 | 1.115 | | 0.152 | 8.204 |  |
|  |  | 53% | 53% | 50% | 0% |  |  |  |  | Recessive | NA | NA | |  |  |  |
| control |  | 106 | 52 | 2 | 0 |  |  |  |  | Homozygous | NA | NA | |  |  | 0.99 |
|  |  | 47% | 47% | 50% | 0% |  |  |  |  | Heterozygous | 1.000 | 1.115 | | 0.152 | 8.204 |  |
|  |  |  |  |  |  |  |  |  |  |  |  |  | |  |  |  |
| rs15927 | ICOSLG, 3UTR | C | CC | CT | TT | 0.792 | 1.072 | 0.639 | 1.799 | Additive | 0.959 | NA | |  |  |  |
| test |  | 61 | 13 | 35 | 12 |  |  |  |  | Dominant | 0.826 | 1.106 | | 0.449 | 2.727 |  |
|  |  | 53% | 54% | 52% | 50% |  |  |  |  | Recessive | 0.811 | 0.896 | | 0.364 | 2.203 |  |
| control |  | 54 | 11 | 32 | 12 |  |  |  |  | Homozygous | 0.773 | 1.182 | | 0.380 | 3.672 | 0.477 |
|  |  | 47% | 46% | 48% | 50% |  |  |  |  | Heterozygous | 0.871 | 1.081 | | 0.424 | 2.753 |  |
|  |  |  |  |  |  |  |  |  |  |  |  |  | |  |  |  |
| rs117576800 | ICOSLG, 3UTR | A | AA | GA | GG | 0.632 | 1.137 | 0.672 | 1.924 | Additive | 0.671 | NA | |  |  |  |
| test |  | 65 | 18 | 29 | 13 |  |  |  |  | Dominant | 0.409 | 1.429 | | 0.611 | 3.339 |  |
|  |  | 55% | 60% | 50% | 54% |  |  |  |  | Recessive | 0.947 | 1.031 | | 0.417 | 2.550 |  |
| control |  | 53 | 12 | 29 | 11 |  |  |  |  | Homozygous | 0.667 | 1.269 | | 0.429 | 3.758 | 0.706 |
|  |  | 45% | 40% | 50% | 46% |  |  |  |  | Heterozygous | 0.373 | 1.500 | | 0.614 | 3.666 |  |
|  |  |  |  |  |  |  |  |  |  |  |  |  | |  |  |  |
| rs11274942 | ICOSLG, 3UTR | CTG | CTG | INS/CTG | INS | 0.560 | 0.853 | 0.501 | 1.454 | Additive | 0.391 | NA | |  |  |  |
| test |  | 69 | 21 | 27 | 12 |  |  |  |  | Dominant | 0.908 | 1.047 | | 0.481 | 2.278 |  |
|  |  | 51% | 54% | 48% | 67% |  |  |  |  | Recessive | 0.208 | 1.958 | | 0.679 | 5.649 |  |
| control |  | 65 | 18 | 29 | 6 |  |  |  |  | Homozygous | 0.362 | 0.583 | | 0.182 | 1.870 | 0.536 |
|  |  | 49% | 46% | 52% | 33% |  |  |  |  | Heterozygous | 0.589 | 1.253 | | 0.552 | 2.843 |  |
|  |  |  |  |  |  |  |  |  |  |  |  |  | |  |  |  |
| rs822335 | PD-L1-promoter | T | TT | TC | CC | 0.268 | 0.735 | 0.426 | 1.269 | Additive | 0.164 | NA | |  |  |  |
| test |  | 71 | 17 | 37 | 6 |  |  |  |  | Dominant | 0.080 | 0.498 | | 0.228 | 1.092 |  |
|  |  | 51% | 43% | 62% | 50% |  |  |  |  | Recessive | 0.793 | 0.852 | | 0.257 | 2.823 |  |
| control |  | 69 | 23 | 23 | 6 |  |  |  |  | Homozygous | 0.646 | 0.739 | | 0.203 | 2.695 | 0.998 |
|  |  | 49% | 58% | 38% | 50% |  |  |  |  | Heterozygous | 0.060 | 0.459 | | 0.203 | 1.038 |  |
|  |  |  |  |  |  |  |  |  |  |  |  |  | |  |  |  |
| rs550207667 | PD-L1-promoter | G | GG | GA | AA | 0.598 | 2.333 | 0.209 | 26.109 | Additive | 0.476 | 2.360 | | 0.208 | 26.803 |  |
| test |  | 119 | 59 | 1 | 0 |  |  |  |  | Dominant | 0.596 | 2.360 | | 0.208 | 26.803 |  |
|  |  | 54% | 54% | 33% | 0% |  |  |  |  | Recessive | NA | NA | |  |  |  |
| control |  | 102 | 50 | 2 | 0 |  |  |  |  | Homozygous | NA | NA | |  |  | 0.990 |
|  |  | 46% | 46% | 67% | 0% |  |  |  |  | Heterozygous | 0.596 | 2.360 | | 0.208 | 26.803 |  |
|  |  |  |  |  |  |  |  |  |  |  |  |  | |  |  |  |
| rs822336 | PD-L1-promoter | G | GG | GC | CC | 0.669 | 0.885 | 0.506 | 1.549 | Additive | 0.648 | NA | |  |  |  |
| test |  | 84 | 28 | 28 | 4 |  |  |  |  | Dominant | 0.465 | 0.766 | | 0.374 | 1.569 |  |
|  |  | 49% | 49% | 55% | 44% |  |  |  |  | Recessive | 1.000 | 0.786 | | 0.200 | 3.081 |  |
| control |  | 87 | 32 | 23 | 5 |  |  |  |  | Homozygous | 1.000 | 1.094 | | 0.267 | 4.476 | 0.956 |
|  |  | 51% | 53% | 45% | 56% |  |  |  |  | Heterozygous | 0.387 | 0.719 | | 0.340 | 1.521 |  |
|  |  |  |  |  |  |  |  |  |  |  |  |  | |  |  |  |
| rs10815225 | PD-L1-promoter | G | GG | GC | CC | 0.066 | 0.377 | 0.128 | 1.106 | Additive | 0.196 | NA | |  |  |  |
| test |  | 104 | 47 | 10 | 1 |  |  |  |  | Dominant | 0.092 | 0.388 | | 0.126 | 1.198 |  |
|  |  | 48% | 46% | 67% | 100% |  |  |  |  | Recessive | 0.492 | NA | |  |  |  |
| control |  | 115 | 55 | 5 | 0 |  |  |  |  | Homozygous | 0.466 | NA | |  |  | 0.945 |
|  |  | 53% | 54% | 33% | 0% |  |  |  |  | Heterozygous | 0.136 | 0.427 | | 0.136 | 1.339 |  |
|  |  |  |  |  |  |  |  |  |  |  |  |  | |  |  |  |
| rs12342381381 | PD-L1-promoter | A | AA | AG | GG | 0.385 | 0.626 | 0.215 | 1.817 | Additive | 0.600 | NA | |  |  |  |
| test |  | 107 | 50 | 7 | 1 |  |  |  |  | Dominant | 0.344 | 0.568 | | 0.174 | 1.851 |  |
|  |  | 48% | 48% | 64% | 50% |  |  |  |  | Recessive | 0.981 | 1.035 | | 0.063 | 16.947 |  |
| control |  | 114 | 55 | 4 | 1 |  |  |  |  | Homozygous | 1.000 | 0.909 | | 0.055 | 14.922 | 0.069 |
|  |  | 52% | 52% | 36% | 50% |  |  |  |  | Heterozygous | **0.012** | NA | |  |  |  |
|  |  |  |  |  |  |  |  |  |  |  |  |  | |  |  |  |
| rs4143815 | PD-L1-3UTR | C | CC | GC | GG | 0.295 | 0.754 | 0.445 | 1.279 | Additive | **0.040** | NA | |  |  |  |
| test |  | 63 | 15 | 33 | 12 |  |  |  |  | Dominant | **0.039** | 0.435 | | 0.196 | 0.966 |  |
|  |  | 50% | 40% | 66% | 48% |  |  |  |  | Recessive | 0.563 | 0.769 | | 0.316 | 1.873 |  |
| control |  | 63 | 23 | 17 | 13 |  |  |  |  | Homozygous | 0.503 | 0.707 | | 0.255 | 1.958 | 0.051 |
|  |  | 50% | 61% | 34% | 52% |  |  |  |  | Heterozygous | **0.013** | 0.336 | | 0.140 | 0.806 |  |
|  |  |  |  |  |  |  |  |  |  |  |  |  | |  |  |  |
| rs141907112 | PD-L1-3UTR | T | TT | TA | AA | 0.186 | NA |  |  | Additive | 0.184 | NA | |  |  |  |
| test |  | 118 | 58 | 2 | 0 |  |  |  |  | Dominant | 0.498 | NA | |  |  |  |
|  |  | 53% | 53% | 100% | 0% |  |  |  |  | Recessive | NA | NA | |  |  |  |
| control |  | 104 | 52 | 0 | 0 |  |  |  |  | Homozygous | NA | NA | |  |  | NA |
|  |  | 47% | 47% | 0% | 0% |  |  |  |  | Heterozygous | 0.498 | NA | |  |  |  |
| HWE: Hardy-Weinberg equilibrium; OR: odds ratio; CI: confidence interval; NA: not applicable | | | | | | | | | | | | | | | | |
